# Diet, Inflammation, and Glycemic Control in Type 2 Diabetes: An Integrative Review of the Literature

**DOI:** 10.1155/2012/542698

**Published:** 2012-12-18

**Authors:** Sarah Y. Nowlin, Marilyn J. Hammer, Gail D'Eramo Melkus

**Affiliations:** College of Nursing, New York University, 726 Broadway, 10th Floor, New York, NY 10003, USA

## Abstract

Type 2 diabetes (T2D) is a growing national health problem affecting 35% of adults ≥20 years of age in the United States. Recently, diabetes has been categorized as an inflammatory disease, sharing many of the adverse outcomes as those reported from cardiovascular disease. Medical nutrition therapy is recommended for the treatment of diabetes; however, these recommendations have not been updated to target the inflammatory component, which can be affected by diet and lifestyle. To assess the current state of evidence for which dietary programs contain the most anti-inflammatory and glycemic control properties for patients with T2D, we conducted an integrative review of the literature. A comprehensive search of the PubMed, CINAHL, Scopus, and Web of Science databases from January 2000 to May 2012 yielded 786 articles. The final 16 studies met the selection criteria including randomized control trials, quasiexperimental, or cross-sectional studies that compared varying diets and measured inflammatory markers. The Mediterranean and DASH diets along with several low-fat diets were associated with lower inflammatory markers. The Mediterranean diet demonstrated the most clinically significant reduction in glycosylated hemoglobin (HbA_1c_). Information on best dietary guidelines for inflammation and glycemic control in individuals with T2D is lacking. Continued research is warranted.

## 1. Background

Thirty-five percent of Americans who are aged 20 years and older have type 2 diabetes (T2D) [1]. The etiology behind such impressive statistics is complex but can be attributed in part to recent changes in the lifestyle choices of Americans, including changes in habitual diet. The What We Eat in America (WWEIA) report, based on data obtained from the National Health and Nutrition Examination Survey (NHANES), demonstrated that grains (mainly refined) have contributed to the bulk of the rise in caloric intake in the US in the last 60 years [2]. Increased consumption of added sugars, processed grains, and saturated fat contributed to excess weight gain, which is an influential risk factor in the development of T2D [3, 4]. In fact, results from implementation of the Diabetes Prevention Program (DPP) illuminated that up to 58% of diabetes can be prevented through lifestyle changes alone, including changes in diet [5].

 One underlying pathophysiological process resulting from poor lifestyle habits is inflammation. Inflammation is present prior to the development of T2D and cardiovascular disease (CVD), contributing to evidence to support the “common soil” hypothesis, which is a reference to the common risk factors for the development of these two diseases [6]. CVD is one of the major complications and causes of death in persons with T2D, supporting this hypothesis [7]. The Multi-Ethnic Study of Atherosclerosis (MESA) showed that higher levels of the inflammatory markers, C-reactive protein (CRP) and interleukin (IL)-6, were associated with an increased risk of developing T2D [8]. Chronic inflammation in T2D initiates with states of obesity, hyperglycemia, insulin resistance, and the overexpression of proinflammatory proteins like CRP and cytokines (IL-1*β*, IL-6, and tumor necrosis factor-alpha (TNF-*α*)) [9]. These proinflammatory molecules activate the cells of innate immunity, which cause damage to tissues in the vasculature, adipose tissue, and pancreas. Acute responses through this proinflammatory pathway are needed for tissue damage repair and can improve function in tissues such as the pancreas, for example; however, chronic expression can lead to pathological changes which result in disease [9]. These changes clinically manifest as CVD, nephropathy, and symptomatic T2D, as the innate immune system attempts to repair the damaged tissue. Moreover, inflammation is stimulated in times of stress, which can be induced by environmental, behavioral, individual, and psychosocial factors [10] and diet-induced hyperglycemia and hypertriglyceridemia [4]. Hyperglycemia and hypertriglyceridemia are significant stressors that have also been shown to cause chronic inflammation and contribute to the pathogenesis of T2D [4].

## 2. Problem and Research Aims

 Although the published literature is populated with articles and systematic reviews demonstrating the relationship between dietary patterns, individual dietary factors, and incidence of diabetes [11, 12], the primary literature linking inflammation to diet and T2D is minimal. One of the few reported areas is with the Mediterranean-style diet, which has been associated with lower inflammatory markers and decreased incidence of T2D [12, 13]. Although several reviews have investigated the effects of diet for T2D control, few disclosed the association with underlying inflammatory responses. Therefore, the aim of this study was to present an integrative review of the published literature on the associations between dietary intake and markers of chronic inflammation in patients with T2D. This review and synthesis of the relevant literature seeks to answer the following research question: what is the current state of the science regarding the association between diet and inflammation in established T2D?

## 3. The Literature Search

 This review targeted adults (≥18 years) diagnosed with T2D. All studies that included a dietary intervention, either exclusively or as part of an intervention, were included. Eligibility criteria included diet, inflammatory markers, and T2D evaluated in randomized clinical trial (RCT), quasiexperimental, or cross-sectional studies. The following databases were searched: PubMed, CINAHL, Scopus, and Web of Science. Keywords used in the search were combinations of the “diet,” “chronic inflammation,” “type 2 diabetes,” “C-reactive protein,” “interleukins,” and “tumor necrosis factor” (TNF). A health sciences librarian was consulted on the selection of these search terms. The search was conducted on articles published between January 2000 (the year of the first appearance of diet, inflammation, and T2D in the literature) and May 2012. Studies were limited to only those written in the English language. Studies that focused on prevention of T2D were excluded, as they did not offer data to assist in the exploration of how diet relates to the inflammatory process within the pathogenesis of T2D. Other exclusion criteria were studies that focused on supplements alone or exercise without an evaluation of, or comparison to, diet.

## 4. Search Outcomes

**Figure 1:**
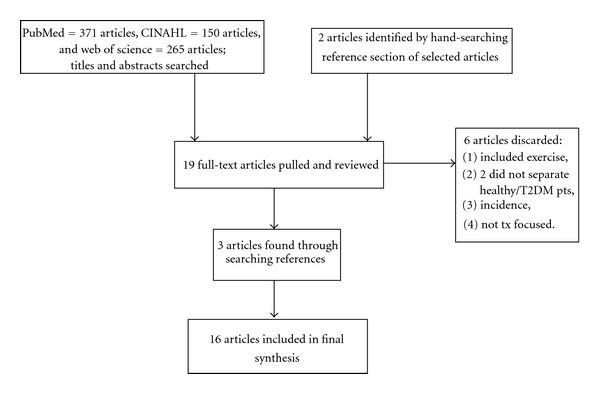
PRISMA flowchart.

The search produced 371 studies in PubMed, 265 studies in Web of Science, and 150 studies in CINAHL (see Figure 1). Titles and abstracts were initially screened. Among these, 19 studies met the full inclusion criteria. The full text from these articles was carefully reviewed. Six more studies were discarded. Two were eliminated because they included predominantly healthy subjects in a cross-sectional study; one was eliminated because it included exercise only, one was eliminated due to its focus on prevention of T2D, and two were eliminated because they focused on acute responses to dietary interventions. Three additional studies were identified by hand-searching reference lists of the selected articles. A final total of 16 papers were included for critical analysis with synthesis of findings. Experimental and nonexperimental studies were included in the analysis. In this integrative review, two studies were cross-sectional [14, 15], two were experimental [16, 17], and twelve were quasiexperimental, as follows: three were crossover experimental designs [18–20], three were pretest-posttest designs with only one group [21–23], and six were pretest-posttest designs with two or more comparison groups [24–29]. More than half of the studies were international with one from the Middle East, one from Canada, three from Australia, and six from Europe. The other five were from the US. All were published in English. All subjects in the cross-sectional studies had a diagnosis of T2D, while several experimental studies utilized healthy and/or obese controls. Acceptable measures for inflammation included serum evaluations of CRP, IL-6, TNF, or a combination of one or more of these with other markers of inflammation.

## 5. Methods

Each study was read twice to ensure identification of salient topics for analysis. Three tables were created to aid synthesis of the data. Table 1 is an overview of information for broad topics created to aid in abstracting those aspects that were definitive in interpreting the implications of the findings. From this table, it became clear that each study reported inflammatory markers at different time points (e.g., some studies did not report baseline values), which was informative for the data reduction process. Quality assessment ratings were established, and each article was scored based on these criteria (Table 2). Tables 3 and 4 contain the results of data abstraction and synthesis, respectively.

### 5.1. Quality Assessment

Articles retrieved for review were assessed for quality of study conduct and reporting [30]. Quality assessment scores were assigned based on 11 questions designed to capture the essence of the study design, methods, and analyses (Table 2). The quality score for each question was assigned based on how well the article fulfilled the expectations. A score of 0 represented absence of the criteria in question, and a score of 1 designated fulfillment of each criterion. These categories included aims, methods (3 questions), diet (3 questions), inflammatory measures, and analysis (2 questions). There was also one question related to results and conclusions with a rating of 0–2, with 2 being of highest quality, which was weighted more heavily due to its importance in interpretation of the study findings. The highest achievable score was 12.

### 5.2. Data Reduction

 Studies were grouped by design method to differentiate results sections for accurate synthesis [30]. Studies were first grouped into experimental (*n* = 14) and correlational (*n* = 2). Studies were then further delineated by diet categories of association with inflammation and those with a focus on diabetes control (glycemic control). Tables 3 and 4 detail these findings. 

## 6. Results

### 6.1. Association between Diet and Inflammation

 Several experimental studies demonstrated a reduction of systemic inflammation following an intervention of a prescribed diet in patients with T2D. Four of the studies with higher quality scores (≥9) demonstrated a significant association between diet and inflammation. Three of these four contained a low-fat (≤30% of energy from fat) dietary component [18, 25, 26]. Azadbakht et al. [18] implemented the Dietary Approaches to Stop Hypertension (DASH) diet in an eight-week crossover intervention study. The DASH diet is characterized by a high intake of whole grains, fruits, and vegetables, while limiting sodium intake (<2400 mg/day) [18]. After controlling for weight reduction, there was a statistically significant decrease in mean CRP in the intervention group compared to the control group (*P* = .03) [18]. The low-fat low-protein diet implemented by Brinkworth et al. [25] is almost identical in macronutrient composition to the DASH diet (see Table 3). After 64 weeks of following this diet with minimal dietary counseling, participants demonstrated a drop in CRP levels, that is comparable to that which was found in the Azadbakht et al. [4] study group, which reached statistical significance (*P* = .04) [25]. The control group in this study received a high-protein, low-fat diet, which also caused a decrease in CRP, and there was not a significant difference in the mean change in CRP between groups (*P* = .61) [25]. Similarly, Davis et al. [26] demonstrated that after following an intervention of either a low-fat or low-carbohydrate diet for 24 weeks, CRP was only found to be significantly reduced in the low-fat diet arm (*P* = .01) and not in the low-carbohydrate arm (*P* = .94). Wolever et al. [29] showed that after 52 weeks of following a low-glycemic-index, low-fat diet (~30% of energy intake from fat), CRP was reduced from baseline by 29% compared to a high-glycemic-index, low-carbohydrate diet that was also high in monounsaturated fat (MUFA) (*P* < .05). Wolever et al. [29] also examined the interaction of diet and time but found no significant effect of time or diet on CRP levels (see Table 1). Of these four studies, only Davis et al. [26] did not report adherence to the prescribed diet. All four studies were controlled for differences in weight loss.

Several other studies also demonstrated reductions in CRP with dietary interventions, albeit with lower quality scores, and with ranges between 4 and 8. Khoo et al. [28] used a crossover design and randomly assigned participants into one of two groups for 8 weeks. One group began on a low-calorie diet and the other a high-protein low-fat diet for eight weeks, then both groups continued on the high-protein low-fat diet for 44 weeks (see Table 1) [28]. Mean-CRP levels were reduced significantly only in the high-protein, low-fat diet at 52 weeks (*P* < .01) [28]. Although diet diaries were collected throughout the study period, adherence to the diet was not reported. Bozzetto et al. [19] conducted a randomized crossover trial with only 12 participants, but the analysis revealed a decreasing mean-CRP level with fasting, 3-hour postprandial, and 6-hour postprandial following 4 weeks of a diet high in MUFAs, compared to the alternate diet which was high in carbohydrates and fiber with a low glycemic index (*P* < .05) [19]. A study by Giannopoulou et al. [27] also demonstrated the anti-inflammatory effects of a diet high in MUFAs (see Table 1). This study was 14 weeks long, and comparison groups included an exercise-only group and a group that was prescribed exercise as well as the high MUFA diet. All three groups demonstrated a significant reduction in mean-CRP at final data collection (*P* < .01); however, there was not a significant difference in this reduction between groups [27]. It is interesting to note here that the diet and exercise group did not have a greater decrease in inflammatory markers than the diet-only group. These results indicate that exercise may not add benefit to improving inflammatory markers in patients with T2D. In another longitudinal study, Barnard et al. [24] randomized 99 participants to receive either a low-fat vegan diet or a conventional American Diabetes Association (ADA) diet for 74 weeks. Results showed a significant reduction of CRP within groups (*P* < .01 for each group) but a nonsignificant difference between the two diet groups [24], perhaps due to the similar macronutrient content of the two dietary interventions. 

Following on the premise that increased fat mass in T2D contributes to inflammation, three other studies implemented diets that were low or very low in energy content [21–23]. Kozłowska et al. [22] implemented a diet with a 20% energy deficit and low protein for eight weeks without a comparison group. This diet did not produce a significant reduction in CRP; however, the 17 participants did exhibit significantly lower TNF-*α* levels (*P* = .002) following the intervention [22]. Additional two studies implemented two weeks of a very-low-calorie diet (VLCD) with obese subjects with T2D in highly controlled hospital environments [21, 23]. Dostlova et al. [21] demonstrated a significant drop in mean-CRP after two weeks of following a VLCD diet (*P* < .05). Mraz et al. [23] found that 2 weeks of a VLCD significantly decreased mean-CRP (*P* < .05), as well as decreased IL-6 levels (*P* < .05). Although significant results were found, such diets cannot be maintained, and as such the long-term effects are not known.

Marfella et al. [16] conducted an RCT in Italy with two groups that were advised to follow a Mediterranean-style diet. The Mediterranean diet is characterized by three servings of fruits and six servings of vegetables or wild leafy greens per day [31]. Fish is consumed 5 to 6 times per week and contributes to the main source of fat, which is monounsaturated and polyunsaturated. The intervention group consumed 4 oz. of red wine per day, while the control group abstained from alcohol. Results revealed significantly greater reductions in CRP (*P* < .01), TNF-*α* (*P* < .01), and IL-6 (*P* < .01) in the intervention group compared to the control group after the 52-week study period [16]. 

In contrast, there were several diets that did not alter inflammatory marker expression significantly. These included the high-carbohydrate/high-fiber/low-glycemic index diet [19], the low-carbohydrate diet implemented by Davis et al. [26], the low-carbohydrate and DPP-style diet implemented by Vetter et al. [17], and the Mediterranean diet [20].

The two cross-sectional studies included in this analysis examined associations between two different aspects of diet and differed significantly in terms of quality in reporting. Åsgård et al. [14] quantified fruit and vegetable intake, while Qi et al. [15] categorized whole grain, germ, and bran intake into quintiles. Qi et al. demonstrated a significant association between CRP and the dietary intake of interest, showing that with increasing quintiles of cereal fiber intake, CRP was significantly lower (*P* for trend = .03) [15]. This inverse relationship was also present between TNF-*α* and cereal fiber intake (*P* for trend = .01). Åsgård et al. [14] also demonstrated a significant inverse correlation between alpha (*α*) and beta (*β*) carotenoids (foods that contain vitamin A) and inflammation (*r* = −.41 and *r* = −.36, resp.).

### 6.2. Diet Influences Glycemic Control

The positive influence of a dietary intervention on glycemic control [16, 20, 21, 24, 27] was examined in five studies. Dostlova et al. [21] measured fasting blood glucose (FBG) as a proxy for glycemic control and demonstrated a significant improvement in FBG following the two-week dietary intervention of a very-low-calorie diet (*P* < .05). Itsiopoulos et al. [20] also demonstrated an improvement in glycemic control following a 24-week crossover intervention with a Cretan Mediterranean diet as evidenced by a significantly reduced mean glycosylated hemoglobin (HbA_1c_) (*P* = .012), compared to the control diet which was the participants' normal eating pattern. Both studies also demonstrated improvement in the homeostatic model assessment (HOMA) score, a measure of insulin resistance (see Table 1), although this change was not statistically significant following the Mediterranean diet [20, 21]. A third study by Barnard et al. [24] found that after following a vegan diet for 72 weeks, subjects had significantly improved HbA_1c_ levels compared to those who followed a conventional diet as recommended by the ADA (*P* = .03) (see Table 3). A Mediterranean diet was also implemented by Marfella et al. [16], along with either four ounces per day of red wine or no alcohol. HbA_1c_ levels were not significantly different between the two groups; however, the mean change in HbA_1c_ was greater than in all other studies included in this review (−1.1% for the intervention group and −1.2% for the control group) [16]. The clinical significance of such a large change in HbA_1c_ will be discussed later. Finally, Giannopoulou et al. [27] reported that although none of the intervention groups demonstrated a significant improvement in HbA_1c_, both the diet-only and diet and exercise groups had a significant reduction in fasting glucose levels (*P* < .05 for both groups). Interestingly, only the two interventions with exercise produced a significant reduction in insulin resistance [27].

## 7. Discussion

 The great variability between studies decreases the ability to draw definitive conclusions about associations between diet, inflammation, and T2D; however, particular themes emerged that are worth noting both from this review and in light of previous research. First, of the ten different diets prescribed that were low in fat, both within and across studies, eight of those diets led to significant reductions in inflammatory markers. These included the DASH diet [18], the low-fat DPP-style diet [26], the low-energy low-protein diet [22], the vegan and ADA diets [24], the high-protein low-fat diet [28], and both the high-protein and low-protein diets [25]. However, this review also supported the hypothesis that the high-MUFA diet decreased inflammation.

Previous reports from the Whitehall and PREDIMED studies indicated that diets high in MUFA and polyunsaturated fatty acids (PUFAs) were correlated with decreased CRP [32, 33] and IL-6 [33], while diets high in saturated fatty acids were positively correlated with CRP [32]. High intake of PUFA was also inversely correlated with incidence of T2D in the Nurse's Health Study [3]. Therefore, it was not surprising that the High-MUFA diet showed anti-inflammatory effects in the diabetic population [16, 19, 20, 29]. Bozzetto et al. [19] lacked baseline values for participants; therefore, it is not appropriate to infer causality for a change in CRP over the 4 weeks of the study period, but rather only in the acute postprandial response. Unfortunately, glycemic control was not concurrently assessed. 

Dietary patterns, as opposed to specific aspects of diet, have also been shown to correlate with levels of chronic inflammatory markers, but this concept was not adequately examined in the selected studies. For example, the western dietary pattern, characterized by a high consumption of sugary drinks, red meat, and poultry and a low consumption of fruits, vegetables, and fiber, was correlated with higher levels of CRP in two large cross-sectional studies conducted in the US [34, 35].

 The impact of diet on the metabolic milieu of T2D is evident from the results of this integrative review and others. However, statistical significance does not necessarily translate into clinical significance or meaningfulness. For example, of the two studies that were able to demonstrate a significantly reduced HbA_1c_ following the prescribed diet, one failed to report baseline values of glycemic control, [20] and the other only demonstrated a mean reduction of 0.4% [24]. While this result is statistically significant, it might not result in improvement in long-term outcomes of T2D, as demonstrated by findings from the United Kingdom Prospective Diabetes Study [36], which showed that a 1% reduction in HbA_1c_ levels can lead to improvements in long-term outcomes and decreased mortality [36]. The implementation of a Mediterranean-style diet and a glass of red wine per day produced a mean decrease in HbA_1c_ of 1.1% (±0.06) [16]. The control group that did not receive the red wine intervention but did follow a Mediterranean diet had a similar decrease in HbA_1c_, as well as CRP, TNF-*α*, and IL-6 [16]. The reductions in HbA_1c_ were not found to be significantly different between intervention and control groups, though the drop in this important glycemic marker is clinically relevant, as it points to decreased incidence of diabetes-related complications and decreased mortality [36]. 

 To further increase their clinical relevance, it is important for studies implementing dietary changes to examine sustainability of the prescribed diet. Although both studies of VLCD reported significant decreases in inflammatory markers, such a diet is not sustainable and is likely harmful for the individuals as evidenced by the significant increase in TNF-*α* following the 2-week VLCD implemented by Mraz et al. [23]. The rationale behind this intensive diet is rapid improvement in metabolic parameters. The lack of long-term followup of the selected patients prevents us from extrapolating from these findings that patients will have sustained results. In fact, the crossover experimental study by Khoo et al. [28] illuminates that after 52 weeks of the prescribed diets, the group that initially received a low-calorie diet did not show a significant decrease in CRP, while the group that was prescribed the high-protein, low-fat diet for all 52 weeks did achieve significant reductions in CRP. This highlights that there may not be merit behind the rapid weight-loss from calorie-restricted programs in the aforementioned studies. 

### 7.1. Gaps in Knowledge in the State of the Science

Due to the limited availability of data for analysis on this topic and the suboptimal quality of reporting within the studies, the results of this integrative review are inconclusive. Many of the studies reviewed suffered from significant methodological shortcomings. First, most of the studies had small sample sizes, with a range between 12 and 162 (mean sample size of 53, and median of 32), limiting the power of the intervention to detect an effect. The two studies with the largest sample sizes implemented the low-glycemic-index diet and Mediterranean diet with wine and showed significant reduction in inflammatory marker expression [16, 29]. Only three studies included in this integrative review conducted a power analysis [18, 24, 29], and one conducted a retrospective power analysis [25], which is paramount in substantiating findings or validating the need for further research if the findings were insignificant. 

Second, several studies did not include transparent definitions of the dietary intervention, rendering it difficult to infer specific dietary factors that decrease inflammation from the results. In previous studies with healthy volunteers, decreased inflammation was correlated with several individual dietary factors including fiber, antioxidant vitamins such as vitamin C and carotenoids, and fruit and vegetable intake. For example, in large prospective studies, whole grain intake has been associated with both decreased inflammatory markers [37] and decreased incidence of T2D [38]. Of the three experimental studies in the current review that indicated whole grains as an aspect of the diet, only one demonstrated decreased CRP levels following the intervention [18]. Similarly, the cross-sectional study examining intake of whole grains showed an inverse association between increasing intake of cereal fiber and CRP levels [15]. However, one study that implemented a high-carbohydrate, high-fiber diet and another that implemented a Cretan-Mediterranean diet, both of which are high in fiber, failed to demonstrate a decrease in CRP following the intervention [19, 20].

A third and final gap in knowledge related to this topic is adherence and change over time. Though several studies included in this study were longitudinal, the longest study was 74 weeks, which does not necessarily indicate that benefits obtained will be sustained in the long-term. This issue was highlighted in the study by Brinkworth et al. [25] in which two low-fat diets, one low-protein, and one high-protein were prescribed to 38 participants with regular dietary counseling for the first 12 weeks. At 12 weeks, HbA_1c_ had decreased by 9.4% (*P* < .001) in both groups [39]. Results in glycemic control were attenuated, however, at 64 weeks due to the fact that participants had not received regular dietary counseling between weeks 12 and 64 [25]. Recidivism such as this will likely be experienced in other long-term studies, underscoring the need for consistent counseling as well as followup in long-term studies that require behavior change [40].

### 7.2. Limitations

There are several limitations of this integrative review. First, the review is limited to the published literature, so it is subject to publication bias. Several authors were contacted via email with requests for unpublished research, but none were available. Second, the articles reviewed for analysis only included a population with diagnosed T2D. This limited the available literature on the influence of diet on inflammation and glycemic control markedly. There is merit in restriction of the search to this population, given that their inflammatory markers will be elevated relative to the general population and would, therefore, benefit from dietary changes in a more clinically significant way. However, this strength may have limited the conclusions in this paper. Finally, it should be noted that this integrative review does not address the mechanism of action underlying reduction of systemic inflammation following a dietary intervention. Discussion of such is beyond the scope of this paper. The review by Visioli et al. [41] concluded that antioxidants, essential fatty acids, and plant extracts have been shown to reduce systemic inflammation. Extrapolating results from this integrative review to make inferences about specific nutrient effects is not possible; however, it can be inferred that nutrient content of the Mediterranean, vegan, and ADA diets may contain more of those qualities mentioned in the review [41]. 

### 7.3. Implication for Future Research

Given the limitations of the findings and the inconclusive results reached by this review, it is clear that more rigorous research is needed. The present level of evidence on this topic is Level III, given that most of the studies were quasiexperimental. This paper, therefore, increases the level of evidence by providing an integrative review of the available research. However, disparate methods of reporting findings have rendered synthesis of extant findings difficult and unnecessarily labor-intensive. Studies should adhere to the transparent reporting standards outlined by the Consolidated Standards of Reporting Trials [42]. Researchers should also focus their efforts on increasing validity and reliability in research conduct. Adherence to dietary interventions needs to be reported and included in analysis of the findings, possibly by including a measure of adherence as a regression variable. Failure to include this critical factor could explain the variability in the findings of this integrative review, for a dose response is a likely influence in the success or failure of an intervention effect. Adequately-powered, high quality studies examining the association between dietary intake and inflammation in T2D are also needed.

Medical nutrition therapy is one aspect of prevention and care of T2D recommended by the ADA. Although the DPP demonstrated a reduction in the incidence of T2D following the adaptation of lifestyle changes, there have been few studies examining the influence of dietary interventions on inflammation and glycemic control in individuals with established T2D in the US. It is important to conduct these studies domestically because of the diversity of the populations affected by this disease and the increasing rates of T2D. For example, further analysis of the results from the DPP revealed an attenuated benefit from the intervention in non-Hispanic Black women, highlighting the need for an understanding of the complex sociocultural factors influencing the African American population [43]. Studies examining the current dietary practices of individuals diagnosed with T2D are necessary, with a particular focus on differences in culturally-bound beliefs surrounding diet, food, and health. 

None of the studies included in this review used a theoretical framework to guide the research, which could have contributed to the disparate findings of the included studies. Theoretical frameworks guide research questions and facilitate the process of asking a complete question. Considering that T2D is both a metabolic and inflammatory disease, it is imperative to design studies that address both of these issues in the methods and the outcome measures. For example, if an intervention of a high MUFA diet is implemented, it may be helpful not only to address inflammatory markers in the outcome measures, but also other influences on inflammation that could confound results such as physical activity, socioeconomic status, environmental stressors, and genetic factors as suggested by Kang's Biobehavioral Model of Stress and Inflammation [10]. It is unclear from the aforementioned studies how exercise, stress, and genetics contributed to the metabolic and inflammatory outcomes measured, and larger, more rigorous studies are needed that control these variables. Finally, based on the results of the aforementioned studies, RCTs using participants with T2D are needed for which the nutrition content of the dietary intervention has been optimized for the treatment of T2D. This includes high intake of fruits and vegetables that are high in flavonoids, which González-Castejón and Rodriguez-Casado [44] report in their review on phytochemicals to have anti-inflammatory and antioxidant activities that may improve the metabolic status of individuals with T2D. The Mediterranean diet is an extremely palatable diet rich in fruits, vegetables, fiber, and olive oil which has been shown with and without the addition of alcohol to improve inflammation, prevent the onset of T2D, and improve glycemic control in established T2D [16, 41, 44]. More high-quality RCTs implementing this diet are needed to provide the evidence base for modifying clinical practice guidelines in medical nutrition therapy for patients with T2D.

## Figures and Tables

**Table 1:** Study methods, details, and outcomes.

Author/year/ country title	Study design and methods	Variables	Instruments	Intervention details	Important methodological flaws/bias noted	Data analysis	Outcomes related to inflammation, glycemic control, and diet
Brinkworth et al. (2004), Australia [25]. Long-term effects of advice to consume a high-protein, low-fat diet rather than a conventional weight-loss diet, in obese adults with type 2 diabetes: one-year followup of a randomized trial.	2-group pretest/posttest design with randomization, longitudinal follow-up. Sample: *N* = 38 obese or overweight adults with type 2 diabetes.	Inflammatory markers: hs-CRP, IL-6, urinary 15-keto-dihyro-PGF. Glycemic control: glucose, HbA_1c_, insulin. Others: body composition, BP, weight, lipids.	Dual X-ray absorptiometry. 24-hour urine collection to measure protein intake from urinary urea : creatinine ratio.	(1) 8 weeks of 30% energy restriction, (2) 4 weeks of energy balance (during first 12 weeks met with dietician every 2 weeks), (3) 52-week maintenance period with minimal counseling (weigh-in every 3 months).	No control for confounding variables (i.e. differences in weight loss, changes in medications over the study period). 39% attrition rate, discussed adequately.	Repeated measures ANOVA. Adjustments for baseline values and body weight with ANCOVA. Regression analysis for relationships between changes in parameters over time.	There was a decrease in CRP for both groups of 14% (*P* = .04), but no difference between groups (*P* = .61). No difference overall in glycemic control at 64 weeks from baseline. Used urinary urea : creatinine ratio to determine adherence to the diet, which was adequate (i.e. higher in high-protein diet group).

Giannopoulou et al. (2005), U.S. [27]. Effects of diet and/or exercise on the adipocytokine and inflammatory cytokine levels of postmenopausal women with type 2 diabetes.	3-group pretest-posttest design with randomization. 1 of 3 interventions: (1) only diet, (2) only exercise, or (3) diet and exercise. The intervention lasted 14 weeks. Sample: *N* = 33 postmenopausal, obese women with type 2 diabetes.	Inflammatory markers: leptin, CRP, adiponectin, TNF-α, and IL-6. All measured by blood samples. Glycemic control: HbA_1c_, fasting blood glucose, insulin. All measured by blood samples. Diet: 24 hr recall every two weeks, but did a postmeal evaluation of markers. Others: body composition (abdominal fat distribution), metabolic data, EKG.	24 hour recalls. Exercise stress test. BodPod used to determine percentage of body fat. MRI for abdominal fat distribution testing.	(1) Diet only: high MUFA Fat 40%, CHO 40%, Protein 20%, Individual modifications made to energy intake based on energy expenditure from exercise and metabolic rate. Initial nutritional counseling conducted at the beginning and at weekly intervals. (2) Exercise only: 60 min walking 3-4 times per week. (3) Combo.	Exercise intervention was “supervised” but not specified how. Subjects completed 24-hr dietary recalls but no explanation given as to what was done with these. No mention of where subjects came from. Only generalizable to postmenopausal obese women with type 2 DM. CRP correlated with weight, BMI, total body fat mass, SAT, and IL-6.	Descriptive data: 2-way ANOVA with post hoc analysis where appropriate. Covariates: total body fat mass, abdominal fat distribution.	For all groups: CRP decreased pre/post with *P* < .01. IL-6 change pre/post was NS for all groups. HbA_1c_ change was NS for all groups. TNF-*α* change was NS for all groups.

Marfella et al. (2006), Italy [16]. Effect of moderate red wine intake on cardiac prognosis after recent acute myocardial infarction of subjects with type 2 diabetes mellitus.	RCT. Sample: 131 participants with type 2 diabetes with recent (<2 months) myocardial infarction (MI), <70 yr, and can drink alcohol. All patients were treated with insulin. Baseline demographics between groups were similar. Subjects randomized to intervention or control group for 52 weeks.	Inflammatory markers: TNF-*α*, IL-6, IL-18, CRP. Glycemic control: insulin sensitivity (HOMA score), insulin, HbA_1c_. Diet: red wine consumption. Others: nitrotyrosine, BMI, lipids, myocardial synchronization, other measures of functional cardiac outcomes.	24-hr dietary recall: at first visit. 4-day food diary: every three months. Echocardiogram.	Both groups advised 2000 kcal/day diet similar to Mediterranean diet. Wine group: 4-oz. glass of red wine each day. Comparison group: no red wine consumption.	Only 115 patients completed the study (8 died). Both groups given dietary advice, which was not a variable in the analysis.	One-way ANOVA for baseline, then Scheffe's test to compare pairs of data. For nonnormally distributed data (IL-6, TNF*α*, IL-18, HOMA, triglycerides), Wilcoxon's test was used. Linear regression used to assess correlations.	Significant higher levels of TNF*α*, IL-6, IL-18, CRP, and nitrotyrosine (*P* < .01 for all) in the control group than wine group. Change in HbA_1c_ was NS different between groups.

Wolever et al. (2008), Canada [29]. The Canadian trial of carbohydrates in diabetes (CCD), a 1-y controlled trial of low-glycemic-index dietary carbohydrate in type 2 diabetes: no effect on glycated hemoglobin but reduction in C-reactive protein.	3-group pre-test/post-test design with randomization. Sample: *N* = 162. Subjects were adults age 35–75 y/o with type 2 diabetes and a BMI of 24–40.	Inflammatory markers: CRP. Glycemic control: insulin sensitivity (HOMA score), fasting glucose, fasting insulin, HbA_1c_. Diet: macronutrient intake. Others: lipid profile, weight, blood pressure, free fatty acids. CRP: log-transformed (those whose statin doses were changed during the study were not included in the analysis of CRP, lipids, and lipoproteins).	3-day food diary records collected 7 times during the 12 month period. Key food diaries from 91% of subjects.	12 months in length. All subjects advised “heart healthy” diet. High-GI diet: avoid low-GI foods, eat low-fat foods. Low-GI diet: exchange high-GI foods for low-GI foods, eat low-fat foods. Low-CHO: decrease SFA intake, increase MUFA intake. Individual dietary counseling provided at 2 and 4 weeks, then every 4 weeks.	No control group.	General linear mixed model. Stats CRP used log-transformed data presented as means and CI's. Time treated as a regression variable. Model covariates: age, BMI, sex, and center that correlated significantly with the response variable. Time × diet: interactions. *data for subjects that were excluded from analysis were included up until the point they were dropped. Considered MAR.	CRP in low-GI diet 29% lower than high-GI diet. CRP was 1.95 (1.68, 2.27) for the low-GI diet, 2.75 (2.33, 3.24) for the high-GI diet, and 2.35 (2.01, 2.75) for the low-CHO diet (*P* < .05 for difference in groups). CRP increased with high-GI diet. HbA_1c _ rose from 6.1 to 6.3%, but between group effect on HbA_1c_ was NS from diet: high GI HbA_1c_ = 6.34 ± 0.05, low GI HbA_1c_ = 6.34 ± 0.05, low GI HbA_1c_ = 6.35 ± 0.05. Significant effect of time on weight, but not significantly different between diet groups.

Barnard et al. (2009), United States [24]. A low-fat vegan diet and a conventional diabetes diet in the treatment of type 2 diabetes: a randomized, controlled, 74-week clinical trial.	2 group pretest-posttest design. Sample: *N* = 99 Subjects were adults with type 2 diabetes. (1) Vegan diet. (2) Conventional ADA diet (2003 guidelines).	Inflammatory markers: CRP. Glycemic control: HbA_1c_, fasting plasma glucose. Others: lipid profile, weight, BMI, waist circumference, albumin, blood pressure. Dietary adherence: vegan-no animal products in 24-h recalls, low fat, low cholesterol conventional-following prescribed energy restrictions, low SFA. Both groups: attended at least 10 of the 22 weekly dietary info sessions.	24 hour recalls ×7. 3-day dietary record ×4. Pedometer. Bouchard 3-day physical activity record.	Initial 1-hr meeting with dietician for each group. Then, 22 weekly group sessions specific to group. Optional sessions every two weeks for the remaining 52 weeks. Medications were adjusted for safety by an endocrinologist.	Not all analyses were performed only using those participants with good adherence. Good adherence not defined. Only CRP measured, was not a primary focus of the study.	Repeated measures ANOVA for HbA_1c_ (as ITT) using time, diet group, and interaction between time × diet with HbA_1c_ as DV. Between subject *t*-tests for all DVs to detect significant changes from baseline to 74 weeks (or until last measurement) and paired-comparison *t*-tests.	After adjustment for medication changes, LDL and non-HDL cholesterol values decreased, and more so in the vegan group. CRP decreased significantly in both groups, but no difference between groups.

Dostlova et al. (2009), Czech Republic [21]. Increased serum concentrations of macrophage inhibitory cytokine-1 in patients with obesity and type 2 diabetes mellitus: the influence of very-low-calorie diet.	Quasi experimental one group with two comparison groups (monitored in the hospital). Sample: total *N* = 54 Group no. 1 (comparison): (*n* = 17) obese, nondiabetic women. Group no. 2 (treatment group): (*n* = 14) obese women w/type 2 DM. Group no. 3 (comparison): (*n* = 23) healthy lean women.	Inflammatory markers: CRP. Glycemic control: glucose, homeostasis model assessment of insulin resistance (HOMA-IR). Others: MIC-1 levels lipids, BMI, body fat composition. Another primary focus of the study was to test levels of macrophage inhibitory cytokine-1 (MIC-1) and mRNA expression of MIC-1 in subcutaneous and visceral fat.	No measure of adherence to diet.	2-week very-low-calorie diet. Only obese women with and without DM were given the IV diet; a 2-week 550 kcal/day diet. All patients were hospitalized.	Highly controlled environment for study. No adverse events reported. Not a sustainable intervention for long-term use. The substudy examining MIC-1 mRNA expression was totally unrelated to the 3 groups receiving the intervention, and these patients were receiving surgery and cannot be compared.	One-way ANOVA followed by post hoc tests. Spearman correlations performed to evaluate the relationship of MIC-1 expression to other variables.	For women with T2DM (*n* = 14), significant changes in the following parameters were observed after the VLCD: BMI, cholesterol, TGs, glucose, insulin, CRP, and HOMA-IR.

Kozłowska et al. (2010), Poland [22]. Adiponectin, resistin, and leptin response to dietary intervention in diabetic nephropathy.	Quasiexperimental one group pretest/posttest design. Sample: *N* = 17 Subjects were obese with T2DM, all took antihypertensives, antidiabetic drugs, phosphate binders, and diuretics. Study duration was 8 weeks.	Inflammatory markers: TNF-*α*, CRP Glycemic control: insulin sensitivity (HOMA score), insulin, HbA_1c_. Diet: not measured. Others: adiponectin, resistin, leptin, total protein, metabolic rate using oxygen and CO_2_ measurements.	DEXA scan for body composition.	Intervention diet: 20% energy deficit 0.8–1 g/kg ideal body weight, 30% cal from fat, 60% cal from CHO. Consult with dietician Q2wk.	No mention of how dietary intake was measured, just reported in a chart (Table 2). No control group. Small sample size, weak design.	Pearson or Spearman's tests for correlations. Repeated measures. Student's *t*-test or Wilcoxon matched pairs.	No correlations between food intake and inflammatory markers made. After intervention, resistin (*P* < .03) and TNF-*α* (*P* = .0023) concentrations were significantly decreased. Resistin concentrations were correlated with TNF (*r* = .51, *P* < .038).

Vetter et al. (2010), U.S. [17]. Effect of a low-carbohydrate diet versus a low-fat calorie-restricted diet on adipokine levels in obese diabetic participants.	Ancillary group in an RCT. Sample: *N* = 79. Participants were obese, with type 2 diabetes, outpatient. (1) Low-carb diet. (2) Low-fat diet.	Inflammatory markers: leptin, adiponectin, TNF-*α*. All measured by blood samples. Glycemic control: HbA_1c_, insulin, fasting blood glucose. All measured by blood samples. Diet: 24 hr recall. Others: weight, BMI, race, age, gender, diabetes related medications, presence of CAD, smoking status.	24-hour recalls. Nutribase Management software to establish micro/macro nutrient content of intake.	Randomly assigned to groups. First month: dietician-led weekly group sessions for each group. Following months: monthly group sessions. Given handouts, sample menus, recipes, etc.	24-hour recall assessed, but not used to assess adherence to the prescribed diet. High attrition rate (54.9%). Demographic variables between those who did and did not complete study were not significantly different. No discussion of how missing data were handled. No difference in macronutrient intake between groups, although it should have been.	Log-transformed data: leptin, adiponectin, and TNF*α*. Repeated measures ANOVA (with time × diet group) used to compare change in weight, adipokines, and dietary intake. Multiple linear regression to describe associations between weight loss and changes in adipokine values.	Inflammatory markers: CRP not measured. TNF-*α*: decrease did not differ with time (*P* = .34) or between groups (*P* = .475). Glycemic control: HbA_1c_: decline in HbA_1c_ was not significantly different between groups (no *P* value provided). Others: weight: declined significantly over time (*P* < .001), however, it was not different between groups (*P* = .181). Leptin: decline did not differ significantly between groups. Adiponectin: increase did not differ significantly between groups Weight loss significantly associated with changes in leptin (*r* = .36) and TNF-*α* (*r* = − .29).

Azadbakht et al. (2011), study conducted in Iran [18]. The Dietary Approaches to Stop Hypertension eating plan affects C-reactive protein, coagulation abnormalities, and hepatic function tests among type 2 diabetic patients.	Crossover intervention design with randomization. Sample: *N* = 31, Convenience sample of adults with type 2 diabetes at the Shaheed Motahari Hospital of Fooladshahr.	Inflammatory markers: CRP. Glycemic control: not measured. Diet: macronutrient intake. Others: ALT, AST, ALP, bilirubin.	3d dietary recall to assess adherence to the prescribed diet. Monthly 3-day physical activity diary.	8-week crossover study with randomization to control or DASH diet. Control diet had fewer PUFA. Energy intake individually tailored. Monthly visits. 4-week washout period.	Unclear how study was conducted, not easy to follow methods. R/o carryover effects. No mention of how long each diet was maintained, if there was a washout period, or why those who had left the protocol left (r/o fatigue effect).	Assessing effect of intervention: paired *t*-test with and without adjustment for weight. Groups compared using a % change calculation. Patients who “deviated from the study protocol” were not included in the analysis (p. 1084).	Patients who received the DASH diet showed a reduction in CRP, plasma fibrinogen, and liver transferase enzymes. These reductions were significant and remained significant after adjusting for the effect of weight.

Bozzetto et al. (2011), Italy [19]. The association of hs-CRP with fasting and postprandial plasma lipids in patients with type 2 diabetes is disrupted by dietary monounsaturated fatty acids.	Preexperimental posttest only Cross-over with randomization (no washout). Sample: 12 participants with type 2 diabetes HbA_1c_ in good control. 4 weeks on each diet. No washout period mentioned.	Inflammatory markers: CRP. Glycemic control: not measured. Diet: not measured. Others: triglyceride-rich lipoproteins (chylomicrons, large VLDL, small VLDL), cholesterol and triglyceride.	Nothing provided in paper.	Diets were isoenergetic: both had kcal of 948 per day. Randomized, crossover design. CHO/fiber/low-GI: C 52%, MUFA 17%, GI 58%. High-MUFA diet: C 45%, MUFA 23%, GI 88%.	No details given concerning dietary advisement. Dietary adherence not measured. Confounding variables not explored. Body weight did not change significantly over the study period, but it should have changed, considering the limited caloric prescription.	Paired *t*-tests to identify differences in outcome measures between the two diets (groups were merged for analysis). Repeated measures ANOVA to evaluate differences in postprandial testing (multiple tests). They claim no carryover effect was detected.	Diet and inflammation: after intervention, fasting CRP values were not significantly different between study diets, although CRP did decrease significantly after the MUFA meal (*P* < .05), not the CHO/fiber meal.

Davis et al. (2011), [26]. Differential effects of low-carbohydrate and low-fat diets on inflammation and endothelial function in diabetes.	2-group pre-test/post-test clinical trial subgroup of larger trial. Sample: subjects were obese with type 2 diabetes with an HbA_1c_ between 6% and 11%. *N* = 51 63% of subjects were Black in both diet groups, in the low-carb group, 11% of the subjects were Hispanic.	Inflammatory markers: CRP, IL-6. Glycemic control: glucose, HbA_1c_. Others: endothelial function (s-ICAM, soluble E-selectin), and reactive hyperemic peripheral arterial tonometry, weight, lipids.	EndoPat to measure peripheral microvascular endothelial function.	Total 24 weeks: subjects randomized to receive either a low-carbohydrate, Atkins-style diet or a low-fat diet (similar to DPP diet). Participants received structured menus for the 1st two weeks. Medications were adjusted based on a predefined algorithm. Diet was reinforced every 6 weeks at scheduled visits.	No control group. No measure of adherence to prescribed diet.	CRP and IL-6 were log-transformed. Unpaired *t*-tests (or Wilcoxon rank) used to detect differences in parameter outcomes between dietary arms. Pearson or Spearman correlations.	Low-fat diet: CRP decreased from 4.0 to 3.0 (*P* = .01). Low-carb diet did not cause a significant change in CRP (*P* = .94). Low-carb diet: sICAM decreased from 234 to 199 (*P* = .001), E-selectin decreased from 92 to 82 (*P* = .05).

Itsiopoulos et al. (2010), Australia [20]. Can the Mediterranean diet lower HbA_1c_ in type 2 diabetes? Results from a randomized cross-over study.	Randomized cross-over (2 groups pretest, posttest). Sample: *N* = 31 Subjects recruited from newspaper had “well-controlled” type 2 diabetes.	Inflammatory markers: CRP, homocysteine. Glycemic control: HOMA, HbA_1c_, fasting glucose. Other: height, age, plasma fatty acids, 24-hr urine albumin and creatinine, weight, waist-hip ratio, intraabdominal adipose tissue (IAAT), BP.	Health and lifestyle questionnaire (not defined). Seven-day diet record (completed by participant). Used changes in plasma levels of several different carotenoids to check compliance with the IV diet.	Intervention was a Mediterranean diet for 12 weeks, then crossover (24 weeks total). 70% of intervention diet meals prepared and provided in bulk for free. Advised to eat 3 servings fruits per day.	No washout period between diet periods. Measurement tools not standardized. Olive oil company provided the olive oil, may lead to biased results. Underpowered.	Data from the two diets were pooled. Repeated-measures ANOVA. Used an interaction term to control for absence of washout period.	Significant reduction in HbA_1c_ in the intervention diet. Trend toward decrease in HOMA and BMI. Increase in plant food intake.

Khoo et al. (2011), Australia [28]. Comparing effects of a low-energy diet and a high-protein low-fat diet on sexual and endothelial function, urinary tract symptoms, and inflammation in obese diabetic men.	2 group pretest-posttest design with randomization. Sample: convenience sample of 31 White men with type 2 diabetes and erectile dysfunction. Randomly assigned to either modified low-calorie diet (LCD) or high-protein low-fat diet (HP diet) for 8 weeks. After first 8 weeks, all subjects were placed on the HP diet for the remaining 44 weeks.	Inflammatory markers: CRP, IL-6, and soluble E-selectin. Glycemic control: quantitative insulin sensitivity check index (QUICKI), glucose, insulin. Diet: compliance, not used in analysis. Others: androgen levels, erectile function, sexual desire, and lower urinary tract symptoms.	Food diaries to monitor compliance. IIEF-5, Sexual Desire Inventory, International Prostate Symptom Scale.	Diet compliance monitored every 2–4 weeks. LCD: 2 liquid meals per day plus small meal for total of 900 kcal/day. HP diet: 300 g lean meat/poultry/fish, 3 servings/day of cereals/bread and low-fat dairy foods, and two fruit and vegetable servings/day. Should induce a 600 kcal deficit from diet. All subjects received menu plans, recipes, and cooking advice. Activity was not restricted.	Adherence not reported or used in analysis, although diet diaries were used. Major focus of the study was sexual and endothelial function. Handling of missing data not discussed.	Maximum likelihood repeated measures mixed models to compare pre/post measures. Used time × diet interaction. Post hoc pairwise tests made.	Inflammation: significant decrease in CRP (at 8 and 52 wk), E-Selectin (at 8 and 52 wk), and IL-6 (at 8 wk only) in HP diet group (*P* < .01), but not LCD group. Glycemic control: insulin sensitivity (QUICKI) significant improved in LCD group, but not HP diet (*P* < .01). Plasma glucose did not improve significantly in either group. Other: significantly weight loss in both groups.

Mraz et al. (2011), Czech Republic [23]. The effect of a very-low-calorie diet on mRNA expression of inflammation-related genes in subcutaneous adipose tissue and peripheral monocytes of obese patients with type 2 diabetes.	Quasiexperimental design with two comparison groups. Sample: *N* = 35 women only. The intervention was only implemented in the 12 obese women with T2DM. Two other control groups: 15 healthy women, and 8 obese women without T2DM. Intervention lasted 2 weeks and was conducted in a hospital setting for close monitoring.	Inflammatory markers: CRP, IL-6, TNF, IL-8, CCL-2. Glycemic control: glucose, HbA_1c_, insulin. Diet: none. Others: subcutaneous adipose tissue for mRNA expression of chemotactic factors, lipid profile, adiponectin, leptin, resistin, weight, BMI, and age.	Nothing provided in paper.	Only obese women with DM were given the IV diet; a 2-week 600 kcal/day diet. All patients were hospitalized.	No adverse events or medication changes reported from the intervention period. Inadequate detail of the intervention. No discussion of possible causes for the abnormal finding of increased TNF following the intervention.	Paired *t*-test or Wilcoxon signed-rank test used to detect differences between measurements pre/post intervention.	TNF-*α* levels were significantly increased in T2DM women after 2 weeks of following the VLCD (*P* < .05 for pre/post Δ). HbA_1c _not assessed after intervention (only 2 weeks). *μ* Δ in CRP pre/post = .94 mg/L, *P* < .05. *μ* Δ in IL-6 pre/post = .8 pg/mL, *P* < .05.

Åsgård et al. (2007), Sweden [14]. High intake of fruit and vegetables is related to low oxidative stress and inflammation in a group of patients with type 2 diabetes.	Cross-sectional design. Sample: 53 participants recruited from newspaper. Type 2 diabetes with HbA_1c_ <10%, 40–75 yr, BMI < 35, and stable body weight ×3 months.	Inflammatory markers: hs-CRP, IL-6, urinary 15-keto-dihyro-PGF. Glycemic control: glucose, HbA_1c_, insulin (baseline descriptive stats only). Diet: fruit and vegetable intake, *α*-tocopherol, γ-tocopherol, ascorbate, *α*-carotene, *β*-carotene, lutein, lycopene. Others: oxidative stress and chromosomal damage markers.	3-day diet recall with precoded instrument that was altered for this study (originally it was for 7 days). Program used to determine micro and macronutrient content from food recalls, name of program not provided.	NA	No limitations section in paper. No adjustments for age or weight. The study participants were similar, but weight higher in females. This participant group answered a newspaper, may be more interested in diet, their diet was in accordance with the Nordic recommendations. Lower mean HbA_1c_ at baseline (<6.2%).	Spearman correlation coefficients with *P* < .01 considered significant. For correlations with inflammation or oxidative stress, significance value was set to <.05. Wilcoxon two sample tests used with non-normally distributed data to determine differences between genders.	IL-6 lower with higher levels of carotenoids (*P* < .01), but not with the larger food groupings of fruits and vegetables. *γ*-tocopherol had positive correlation with CRP.

Qi et al. (2006), U.S. [15]. Whole-grain, bran, and cereal fiber intakes and markers of systemic inflammation in diabetic women.	Nested cohort in longitudinal analytic study of the Nurses' Health Study. Sample: *N* = 902 Women between 30–55 with type 2 diabetes as defined by National Diabetes Data group (diagnosis made prior to 1997).	Inflammatory markers: CRP, TNF-R2, Glycemic control: not assessed. Diet: glycemic load, glycemic index, quintiles of increasing intake of whole grains, bran, cereal fiber, total fiber and germ Q1–Q5. Others: endothelial function (s-ICAM, soluble E-selectin) oxidative stress and chromosomal damage markers.	Semiquantitative food frequency questionnaire. Questionnaire from the Nurse's Health Study to collect anthropometric and lifestyle data.	No intervention.	“Whole grains were previously described” but the paper referred to was written by different authors and was in a study about men. No limitations section. Data collected between 1989-1990. Only women included.	Associations evaluated with linear regression. Intake of dietary variables assigned to quintiles. Inflammatory values log-transformed and median value assigned for each of the 5 quintiles. Adjusted for age, BMI, smoking, alcohol, physical activity, aspirin use, HbA_1c_, dx of hypertension or hypercholesterolemia, postmenopausal hormone use, glycemic index and magnesium.	Women with higher intakes of whole grains, bran, and cereal fiber had lower levels of CRP and TNF after adjusting for age, BMI, lifestyle, and dietary covariates.

**Table 2:** Quality Assessment tool and scores.

Article	Aims	Methods		Diet of interest			Recruitment	Inflammation	Confounding variables	Missing	Conclusions	Quality rating
	(1 pt)	(1 pt each)		well-defined? (1 pt each)			(1 pt)	(1 pt)	(1 pt)	data/attrition (1 pt)	(2 pts)	(out of 12 pts)
1st Author	Clearly described	Read	Repr	Ad	TO	Diet	Discussed	Measures appropriate	Controlled	Properly handled	Appropriate	Total

Åsgård	1	0	1	1	0	0	1	1	0	1	1	7
Itsiopoulos	1	1	0	1	1	1	1	1	0	0	0	7
Davis	1	1	0	1	1	0	1	1	1	0	2	9
Qi	1	1	0	1	1	1	0	1	1	0	2	8
Dostlova	1	0	0	1	0	0	0	1	1	0	1	5
Azadbakht	1	1	0	1	1	1	1	1	1	0	2	10
Bozzetto	1	0	0	0	0	1	1	1	0	0	2	6
Giannopoulou	1	1	0	0	1	1	0	1	1	0	2	8
Barnard	0	1	0	1	1	1	1	1	0	1	1	8
Wolever	1	1	0	1	1	1	0	1	1	1	1	9
Vetter	0	1	0	0	1	0	0	0	1	0	1	4
Kosłowska	1	0	0	0	1	0	0	1	0	0	1	4
Marfella	1	1	0	0	1	0	0	1	0	0	1	5
Khoo	1	1	0	1	1	0	1	1	0	0	1	7
Mraz	1	0	0	0	1	0	0	1	0	0	1	4
Brinkworth	1	1	1	1	1	1	1	1	0	1	1	10

Note. Ad: adherence; Diet: dietary intervention defined; Read: readability; Repr: reproducibility; TO: appropriate use of temporal ordering.

**Table 3:** Experimental studies: characteristics and results of data abstraction.

Author/year/ country	Design	Study length (week)	Subjects	Dietary intervention		Outcomes			Quality rating
				Diet details	Control for weight loss differences?	ΔCRP	ΔHbA_1c_%	Other
Brinkworth et al. (2004), Australia [25]	2 group pretest/posttest comparison design with randomization	64	*N* = 38 F = 61% M = 39% *μ*age = 61.8	Low-protein (15%) CHO 55% Fat 30% versus High-protein (30%) CHO 40% Fat 30%	Yes Weight	*P* = .61 difference between diets, but overall decrease *P* = .04 Low-protein: pre: 4.2 post (64 wk): 3.6 High-protein: pre: 5.0 post (64 wk): 3.8	*P* = .38* Low-protein: pre: 6.2 post (64 wk): 6.6 High-protein: pre: 6.5 post (64 wk): 6.6	NA	10

Giannopoulou et al. (2005), U.S. [27]	3-group, pretest-posttest with randomization	14	*N* = 33 Female only *μ*age= 57	Diet only CHO 40% Fat 40% (MUFA 30%) Exercise only Diet and exercise	No	*P* < .01 for all three groups No raw data available	NS Δ	NS Δ in TNF-*α* or IL-6 for all three groups	8

Marfella et al. (2006), Italy [16]	RCT	52	*N* = 115 M/F data not given *μ*age = 35.8	Wine: 4 oz. per day + Mediterranean diet Control group: no wine + Mediterranean diet	No	*P* < .01* Changes in raw CRP values not available	*P* = NS* Changes in HbA_1c_ Wine: −1.1 ± 0.06 Control: −1.2 ± 0.7	TNF-*α* *P* < .01* Changes in raw TNF values not available IL-6 *P* < .01* Changes in raw IL-6 values not available	5

Wolever et al. (2008), Canada [29]	3 groups, pretest-posttest with randomization	52	*N* = 162 F = 54% M = 46% *μ*age = 59.9	High GI Low GI Low CHO CHO 20%–25% Fat not provided, but high MUFA in Low-CHO diet	Yes BMI	Low GI-diet: *P* < .05 (different in *μ*CRP postintervention between low and high GI diets) postintervention *μ*: High GI: 2.75 Low GI: 1.95 Low CHO: 2.35	NS difference between groups, HbA_1c_ rose during intervention High GI: 6.34 Low GI: 6.34 Low CHO: 6.35	NA	9

Barnard et al. (2009), U.S. [24]	2 groups, pretest-posttest	74	*N* = 99 F = 61% M = 39% *μ*age = 55.7	Vegan diet CHO 75% Fat 10% Conventional ADA diet C 60–70% Fat < 7% saturated fat	Yes Weight change	*P* = .65* Vegan: (*P* < .01) −1.9 ± 0.6. Conventional: (*P* < .01) −2.4 ± 0.13	*P* = .03* (only using data prior to median adjustments) Vegan: (*P* < .01) −0.4 ± 0.14 Conventional: (*P* = NS) 0.01 ± 0.8	*P* = .01 Total cholesterol decreased more in vegan group	8

Dostlova et al. (2009), Czech Republic [21]	Quasi-experimental one group with two comparison groups (monitored in the hospital)	2	T2DM group *n* = 14 F only *μ*age = 56.1	Very-low-calorie diet (550 kcal/day)	No	*P* < .05 (Δ in *μ*CRP pre: 13.2 ± 3.5/post: 7.1 ± 2.4)	Fasting glucose *P* < .05 (Δ in *μ*fasting glucose pre/post)	HOMA: *P* < .05	5

Kozłowska et al. (2010), Poland [22]	Quasi-experimental (one-group pretest-posttest)	8	*N* = 17 F = 41% M = 59% *μ*age = 66.6	Low energy/Low protein diet (20% energy deficit, 0.8–1.0 g/kg) CHO 60% F 30%	No	*P* = .778 (Δ in *μ*CRP pre/post intervention) pre: 2.6 (0.4–13.9) post: 3.4 (0.3–12.4)	*P* = .485 (Δ in *μ*HbA_1c_ pre/post intervention) pre: 7.7 ± 1.4 post: 7.3 ± 1.4	*P* = .002 (Δ in *μ*TNF pre/post intervention) pre: 10.6 ± 7.5 post: 7.8 ± 5.1	4

Vetter et al. (2010), U.S. [17]	RCT subgroup	26	*N* = 79 F = 11% M = 89% *μ*age = 59.7	Low-carb CHO < 30 g/day versus Low-fat (DPP diet) Fat < 30% + 500 kcal/day deficit	No	Not measured	*P* = NS* Changes in HbA_1c_ Low-carb: −0.6 (1.2) Low-fat: −0.1 (1.2)	TNF-*α* *P* = NS* Changes in TNF Low-carb: −1.5 (7.1) Low-fat: −1.5 (5.6)	4

Azadbakht et al. (2011), study conducted in Iran. [18]	Cross-over with randomization (with 4 week washout period)	8	*N* = 31 F = 59% M = 41% *μ*age = 55	DASH diet CHO 50–60% Fat < 30% *High in whole grains, fruits, vegetables. Low sodium versus control diet	Yes Weight change	*P* = .03* DASH: pre: 2.9 ± 0.31 post: 2.03 ± 0.27 Control: pre: 3.11 ± 0.30 post: 2.92 ± 0.20	No measure of glycemic control	Fibrinogen: *P* = .001* DASH: *μ* change: 38.7 Control: *μ* change: 140	10

Bozzetto et al. (2011), Italy [19]	Preexperimental posttest only. Cross-over with randomization (no washout)	4	*N* = 12 F = 25% M = 75% *μ*age = 59	High-carb/high-fiber/low GI CHO 52% MUFA 17% *GI 58% versus High-MUFA diet CHO 45% MUFA 23% *GI 88%	No	MUFA meal: *P* < .05 (Δ in *μ*CRP when compared with fasting CRP) Fasting: 2.11 ± 2.02 3 h: 1.98 ± 1.99 6 h: 2 ± 2.06 NS Δ in *μ*CRP after CHO/fiber meal	No measure of glycemic control	NA	6

Davis et al. (2011), U.S. [26]	2 groups pretest-posttest with randomization subgroup of larger trial	24	*N* = 51 F = 76% M = 24% *μ*age = 54.5	Low-fat (DPP diet) Fat < 25% versus Low-CHO (Atkins diet) CHO < 20%	Yes, weight loss equal between groups	Low-fat: *P* = .01 Δ in *μ*CRP pre: 4.0 ± 0.77 post: 3.0 ± 0.77 Low-CHO: *P* = .94 Δ in *μ*CRP pre: 3.1 ± 0.42 post: 3.6 ± 0.68	*P* = .72 ns *μ*HbA_1c_ reduced by 0.18 ± 0.16 for pooled groups	IL-6 ns *μ*Wt loss: 11 lb. *P* = .72 for pooled groups	9

Itsiopoulos et al. (2010), Australia [20]	Cross-over with randomization (no washout)	24	*N* = 27 F = 41% M = 16% *μ*age = 59	Mediterranean Diet: CHO 44% Fat 40% (>50% MUFA) versus Control diet: ad lib.	No	*P* = .576* Med diet: 2.38 [1.66, 3.10] Control: 2.49 [1.69, 3.30]	*P* = .012* Med diet: 6.8 [6.3, 7.3] Control: 7.1 [6.5, 7.7]	HOMA, *P* = .061* Med diet: 5.2 (3.9, 6.6) Control: 6.1 (4.4, 7.8)	7

Khoo et al. (2011), Australia [28]	2 groups pretest-posttest with randomization	52	*N* = 31 M only *μ*age = 60.2	Low-calorie diet 900 kcal/day versus High-protein, low-fat Fat < 30% + 600 kcal/day deficit	No	Low-calorie: NS pre: 3.82 ± 1 post (52 weeks): 3.79 ± 0.82 High-protein: *P* < .01 pre: 8.32 ± 1.29 post (52 weeks): 2.85 ± 1.01	Not provided, change in plasma glucose was ns.	IL-6 Low-calorie: *P* = NS pre: 1.59 ± 0.29 post (52 weeks): 1.7 ± 0.42 High-protein: *P* = NS pre: 3.28 ± 0.37 post (52 weeks): 2.4 ± 0.52 (*P* < .05 group and time effect)	7

Mraz et al. (2011), Czech Republic [23]	Quasi-experimental design with two comparison groups	2	*N* = 35 F only *μ*age = 65.6	Very-low-calorie diet 600 kcal/day Healthy and obese subjects did not receive diet	No	*μ* Δ in CRP pre/post = 0.94 mg/L *P* < .05 Raw data not provided	Not provided	*μ* Δ in IL-6 pre/post = 0.8 pg/mL *P* < .05	4

Note: CRP: C-reactive protein; HbA_1c_: glycosylated hemoglobin; F: females; M: males; μ: mean; μage: mean age; Δ: change in; HOMA: homeostatic model assessment; DPP: Diabetes Prevention Program; DASH: Dietary Approaches to Stop Hypertension; GI: Glycemic Index; MUFA: monounsaturated fatty acid; ns: not significant; C: energy from carbohydrates; F: energy from fat; *: Significance of between group differences.

**Table 4:** Cross-sectional studies: characteristics and results of data abstraction.

Author/yr/country	Design	Dietary assessment	Subjects	Dietary component of interest	Measurements			Quality rating
					CRP	IL-6	TNF
Åsgård et al., (2007), Sweden [14]	Crosssectional	3-day dietary survey; precoded	*N* = 54 F = 53% M = 47% *μ*age = 62.6	Fruit and vegetable intake	*P* = .02 (positive correlation with *γ*-tocopherol) *r* = .31	*P* < .01 (negative correlation with plasma carotenoids) *α*-carotenoids: *r* = −.41 *β*-carotenoids: *r* = −.36	NA	7

Qi et al. (2006), U.S. [15]	Crosssection of a cohort in prospective longitudinal study	Semiquant FFQ	*N* = 902 Females, no men in study *μ*age ~ 58.5	Whole grains, germ, and bran	*P* = .03 (*P* for negative trend of CRP w/increasing quintiles of cereal fiber intake)	NA	*P* = .01 (*P* for negative trend of TNF w/increasing quintiles of cereal fiber intake)	8

Note: CRP: C-reactive protein; IL-6: interleukin-6; TNF: tumor necrosis factor-alpha; F: females; M: males; *μ*age: mean age; Semiquant: semiquantitative; FFQ: food frequency questionnaire; BMI: body mass index.
